# Schistosoma mansoni Larvae Do Not Expand or Activate Foxp3^+^ Regulatory T Cells during Their Migratory Phase

**DOI:** 10.1128/IAI.00408-15

**Published:** 2015-09-10

**Authors:** Stephen A. Redpath, Nienke van der Werf, Andrew S. MacDonald, Rick M. Maizels, Matthew D. Taylor

**Affiliations:** a Institute of Immunology and Infection Research, School of Biological Sciences, University of Edinburgh, Edinburgh, United Kingdom; b Centre for Immunity, Infection and Evolution, School of Biological Sciences, University of Edinburgh, Edinburgh, United Kingdom

## Abstract

Foxp3^+^ regulatory T (Treg) cells play a key role in suppression of immune responses during parasitic helminth infection, both by controlling damaging immunopathology and by inhibiting protective immunity. During the patent phase of Schistosoma mansoni infection, Foxp3^+^ Treg cells are activated and suppress egg-elicited Th2 responses, but little is known of their induction and role during the early prepatent larval stage of infection. We quantified Foxp3^+^ Treg cell responses during the first 3 weeks of murine S. mansoni infection in C57BL/6 mice, a time when larval parasites migrate from the skin and transit the lungs en route to the hepatic and mesenteric vasculature. In contrast to other helminth infections, S. mansoni did not elicit a Foxp3^+^ Treg cell response during this early phase of infection. We found that the numbers and proportions of Foxp3^+^ Treg cells remained unchanged in the lungs, draining lymph nodes, and spleens of infected mice. There was no increase in the activation status of Foxp3^+^ Treg cells upon infection as assessed by their expression of CD25, Foxp3, and Helios. Furthermore, infection failed to induce Foxp3^+^ Treg cells to produce the suppressive cytokine interleukin 10 (IL-10). Instead, only CD4^+^ Foxp3^−^ IL-4^+^ Th2 cells showed increased IL-10 production upon infection. These data indicate that Foxp3^+^ Treg cells do not play a prominent role in regulating immunity to S. mansoni larvae and that the character of the initial immune response invoked by S. mansoni parasites contrasts with the responses to other parasitic helminth infections that promote rapid Foxp3^+^ Treg cell responses.

## INTRODUCTION

A hallmark of parasitic helminths is their ability to persist for years within their host despite constant pressure from the immune system. To achieve this, helminths subvert the host immune system by hijacking the regulatory networks that keep it in check (1, 2). Foxp3^+^ regulatory T (Treg) cells are a principal component of this network and are potent suppressors of immunity (3). As such, they are a key cell type targeted by helminths in defense against attack from the host immune system (4). The expansion and activation of Foxp3^+^ Treg cells occur within the first week of both filarial (5–7) and intestinal (8–10) nematode infections. This early induction of Foxp3^+^ Treg cells impairs late-stage effector immunity, to the detriment of host protection (7, 8, 11). Thus, nematode infections bias early immune responses toward regulation to benefit their own survival.

Schistosoma mansoni is a blood-dwelling trematode parasite that is the etiological agent of the tropical disease hepatic schistosomiasis (12). Infective S. mansoni cercariae penetrate the skin of their host and migrate via the circulation, transiting the lungs to reside as adults in the mesenteric veins, where they mate and lay eggs (12). Infections of this type are typically chronic, and the liver fibrosis, portal hypertension, and intestinal bleeding that characterize the disease arise as a consequence of the host immune response to the parasite's eggs (13). During the patent, egg-producing phase of disease (week 5 onwards), Foxp3^+^ Treg cells are activated and suppress Th2 responses, controlling immunopathology in the liver (14–16) and in the colon (17). However, little is known of their role and induction in the early larval lung transit phase of disease.

Although the protective immune mechanisms underlying resistance to S. mansoni larvae in primary infections are poorly understood, during challenge infections, it has been shown that immune responses directed against lung-stage S. mansoni larvae are required for protection (18, 19). Protective immunity is significantly elevated in the absence of the suppressive cytokine interleukin 10 (IL-10) (20, 21), suggesting that immunity to S. mansoni larvae in the lung is inhibited by immune regulation. IL-6 deficiency leads to enhanced Th2 responses and increased protective immunity to lung-stage S. mansoni larvae (22), and the absence of IL-6 can impair Foxp3^+^ Treg cell function during Heligmosomoides polygyrus infection, resulting in increased Th2 effector responses and parasite killing (23). These data suggest a role for Foxp3^+^ Treg cells in the suppression of protective Th2 responses to S. mansoni larvae in the lungs, potentially via IL-10.

We hypothesized that larval S. mansoni parasites rapidly co-opt Foxp3^+^ Treg cell function at an early stage of infection to benefit their own survival, inducing the activation and expansion of Foxp3^+^ Treg cells during the period when the larvae are most vulnerable to immune attack. However, we found that S. mansoni larvae do not induce a Foxp3^+^ Treg cell response during the early stage of infection in C57BL/6 mice. During the first 3 weeks of infection, there was no expansion in the proportions or numbers of Foxp3^+^ Treg cells in the lymph nodes (LN) draining the skin inoculation site, the lungs, the lung-draining LN, or the spleen. Furthermore, Foxp3^+^ Treg cells at these sites did not exhibit an increase in activation status in response to infection, as their expression levels of CD25, Foxp3, and Helios remained constant. Infection also failed to stimulate the production of IL-10 by Foxp3^+^ Treg cells, despite increased IL-10 production by CD4^+^ Foxp3^−^ IL-4^+^ Th2 cells. These data suggest that, in contrast to the responses induced by other helminth infections (5–10, 24, 25), S. mansoni infection does not induce a Foxp3^+^ Treg cell response in the initial stages of infection.

## MATERIALS AND METHODS

### Animals, infections, and parasites.

Female C57BL/6 mice were bred in-house and maintained under specific-pathogen-free conditions at the University of Edinburgh. Mice were used at 6 to 8 weeks of age, and all animal work was conducted in accordance with the Animals (Scientific Procedures) Act 1986. Biomphalaria glabrata snails infected with S. mansoni parasites were obtained from F. Lewis (Biomedical Research Institute, Rockville, MD). C57BL/6 mice were infected percutaneously with 70 S. mansoni cercariae. S. mansoni parasite numbers were quantified by portal perfusion at weeks 2 and 3 to confirm successful infections. A count of 10.4 ± 5.4 (mean ± standard deviation) parasites was recovered at week 2, and a count of 10.6 ± 7.9 was recovered at week 3.

### Cell purifications and *in vitro* stimulation.

The lungs, lung-draining thoracic lymph nodes (tLN), inguinal lymph nodes (iLN) draining the abdominal skin, and spleens were collected. The tLN, iLN, and spleens were dissociated to obtain a single-cell suspension in RPMI 1640 (Invitrogen) supplemented with 100 U/ml penicillin, 100 μg/ml streptomycin, 2 mM l-glutamine, and 5% fetal calf serum (FCS). To isolate lung lymphocytes, lung lobes were dissociated in RPMI 1640 with 5% FCS (Invitrogen), 100 U/ml penicillin–100 μg/ml streptomycin, and 2 mM l-glutamine and then incubated in 250 μg/ml collagenase D (Roche) and 10 μg/ml DNase (Roche) for 30 min at 37°C before being passed through a 70-μm nylon mesh. Erythrocytes in spleen and lung preparations were lysed using red blood cell lysis buffer (Sigma). For measurement of intracellular cytokines, cells were stimulated for 4 h with 0.5 μg/ml phorbol myristate acetate and 1 μg/ml ionomycin, with 10 μg/ml brefeldin A added for the final 2 h (Sigma-Aldrich).

### Flow cytometry.

The following antibodies were used: Alexa Fluor 700-conjugated anti-CD4 antibody (RM4-5; BD Bioscience), fluorescein isothiocyanate-conjugated or allophycocyanin-conjugated anti-Foxp3 antibody (FJK-16S; eBioscience), phycoerythrin-conjugated anti-Helios antibody (22F6; Biolegend), phycoerythrin-conjugated anti-CD25 antibody (PC61 5.3; Invitrogen), phycoerythrin-conjugated anti-IL-4 antibody (11B11), Alexa Fluor 647-conjugated anti-IL-13 antibody (ebio13A; eBioscience), Pacific blue-conjugated anti-T cell receptor β (TCR-β) antibody (H57-597; Biolegend), allophycocyanin-conjugated anti-IL-10 antibody (JES5-16E3; eBioscience), and fluorescein isothiocyanate-conjugated anti-gamma interferon (IFN-γ) antibody (XMG1.2; BD Bioscience). Nonspecific binding was blocked with 4 μg of rat IgG per 1 × 10^6^ cells. Intracellular staining for Foxp3 and Helios was performed using a Foxp3-staining buffer kit (eBioscience). For intracellular cytokine staining, dead cells were excluded using the Live/Dead aqua dead cell stain kit (Molecular Probes), and cells were fixed and permeabilized using the BD Cytofix/Cytoperm kit. Flow cytometry was performed using a FACSCanto 2 or an LSR 2 (BD Biosciences), running FACSDiva software (BD Biosciences). Analysis was performed using FlowJo (Tree Star).

### Statistics.

Statistical analysis was performed using JMP (SAS) version 8. Parametric analysis of combined data from multiple-repeat experiments or experiments containing more than two groups was performed using analysis of variance (ANOVA), followed by the least squares means (LSM) Student's *t* test.

## RESULTS

### Transit of S. mansoni larvae through the lung does not cause expansion of Foxp3^+^ Treg cells.

**FIG 1:**
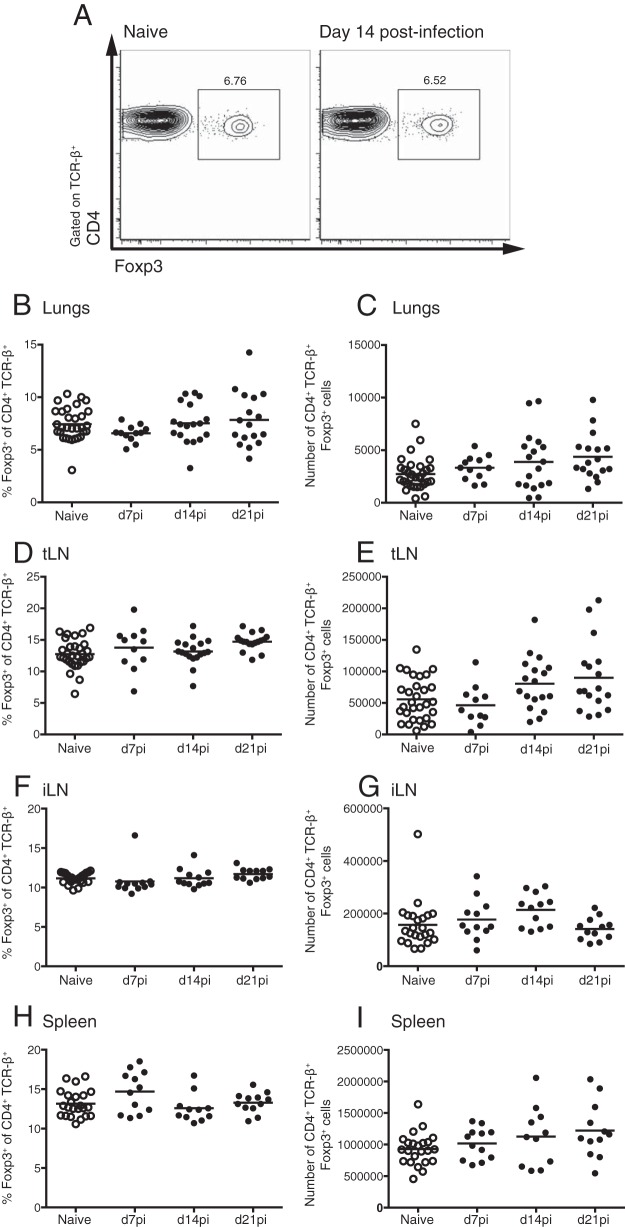
S. mansoni larval migration does not expand Foxp3^+^ Treg cells. C57BL/6 mice were infected with 70 S. mansoni cercariae, and CD4^+^ Foxp3^+^ Treg cells were measured in the lungs, tLN, iLN, and spleen at days 7, 14, and 21 of infection. (A) Representative CD4 and Foxp3 staining of lung CD4^+^ TCR-β^+^ T cells. (B to I) Percentages and numbers of CD4^+^ TCR-β^+^ Foxp3^+^ cells within the lungs (B and C), tLN (D and E), iLN (F and G), and spleen (H and I). Panels show combined data from 3 independent experiments with 4 to 6 mice per group. Symbols represent individual mice, and bars show mean values. Open circles denote results for naive mice, and closed circles denote results for S. mansoni-infected mice.

The rapid recruitment and activation of Foxp3^+^ Treg cells is a feature of infections with parasitic nematodes (7–9, 14, 26). However, the role of Foxp3^+^ Treg cells in immunity to early-stage S. mansoni infection is unclear. To address whether Foxp3^+^ Treg cell responses are evoked during the initial stages of S. mansoni infection, as the larvae transit from the skin through the lungs to the hepatic portal and mesenteric vasculature, we infected C57BL/6 mice with 70 S. mansoni cercariae and measured Foxp3 responses in the iLN (draining the skin site of inoculation), lungs, tLN (draining the lungs), and spleen at days 7, 14, and 21 postinfection (p.i.). Throughout this period, there was no difference in the percentages of CD4^+^ T cells expressing Foxp3 in the lungs in naive and S. mansoni-infected wild-type mice (Fig. 1A and B). Although there was a trend for an increase in the absolute numbers of Foxp3^+^ cells in the lungs of infected mice, this did not reach statistical significance (Fig. 1C). Similarly, the percentages and numbers of Foxp3^+^ CD4^+^ T cells remained unchanged by S. mansoni infection in the tLN, iLN, and spleen (Fig. 1D to I). Therefore, in contrast to the case for other helminth infections, early-stage S. mansoni infection does not induce the expansion of Foxp3^+^ Treg cells in the infection site, draining LN, or spleen.

### Foxp3^+^ Treg cells are not activated by migration of S. mansoni larvae.

**FIG 2:**
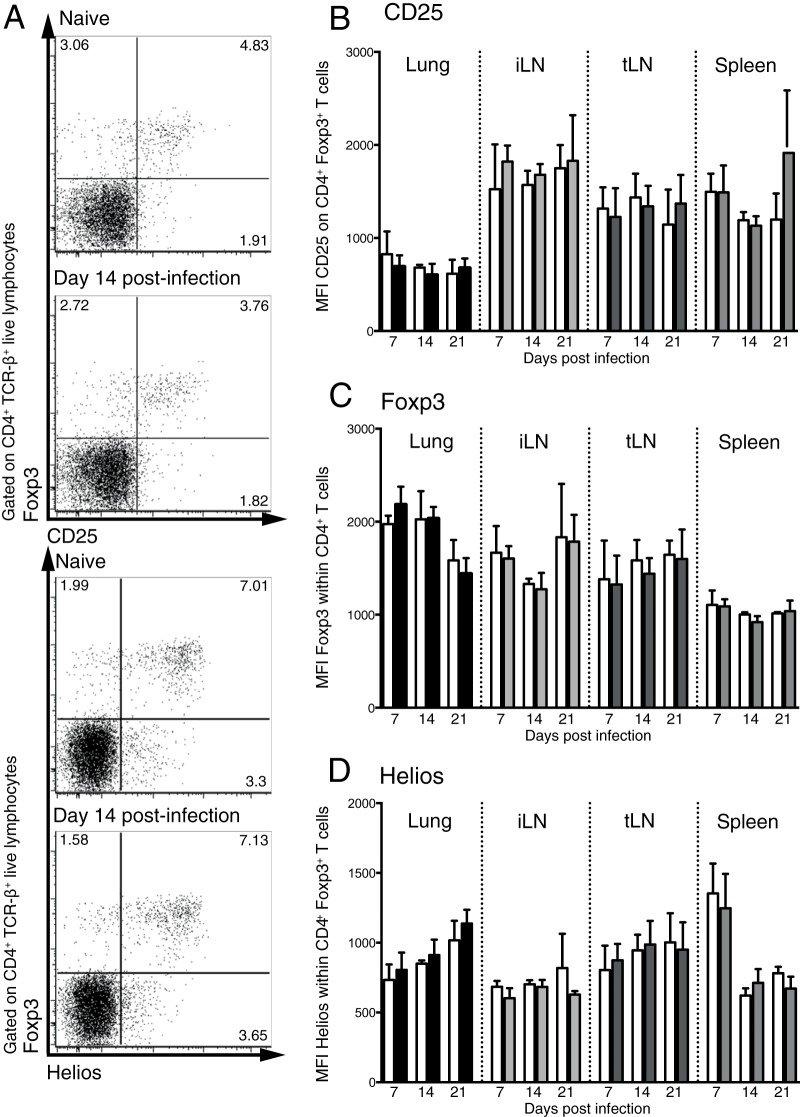
S. mansoni larval migration does not increase the activation level of Foxp3^+^ Treg cells. C57BL/6 mice were infected with 70 S. mansoni cercariae, and the expression of CD25, Foxp3, and Helios was quantified on CD4^+^ TCR-β^+^ Foxp3^+^ Treg cells in the lungs, iLN, tLN, and spleen on days 7, 14, and 21 p.i. (A) Representative staining showing Foxp3 versus CD25 and Helios in the lungs at day 14 p.i. (B to D) Bar graphs showing MFI of CD25 (B), Foxp3 (C), and Helios (D) on CD4^+^ TCR-β^+^ Foxp3^+^ Treg cells. Panels are representative of three separate experiments with 4 to 6 mice per group. Bars show mean values, and error bars show standard deviations. Open bars denote results for naive mice, and shaded bars denote results for S. mansoni-infected mice.

Although the proportion of Foxp3^+^ Treg cells was unaltered during the larval stage of S. mansoni infection (Fig. 1), it is possible that the Treg cells were in a heightened state of activation. To determine whether Foxp3^+^ Treg cells were activated by S. mansoni larvae, we measured the expression of CD25, Foxp3, and Helios on Foxp3^+^ T cells in the lungs, tLN, iLN, and spleen during the first 3 weeks of S. mansoni infection (Fig. 2A). While Helios expression was originally reported to distinguish thymic Treg cells from peripheral Treg cells (27), more recent evidence suggests that it is a marker of Treg cell activation (28). In contrast to the egg phase of infection, when Foxp3^+^ Treg cells actively control pathology (14–17), there was no significant increase in the mean fluorescence intensity (MFI) of CD25 expression on Foxp3^+^ Treg cells in the lungs or at any other site measured during early-stage S. mansoni infection (Fig. 2B). Similarly, there was no increase in Foxp3 MFI iLN, or spleen during the first 3 weeks of infection (Fig. 2C). The expression of the transcription factor Helios also remained constant following infection (Fig. 2D). Taken together, these data suggest that the migration of S. mansoni larvae through the lungs does not significantly alter the activation state of Foxp3^+^ Treg cells.

### Foxp3^+^ Treg cells are not a major T cell source of IL-10 in the early stage of S. mansoni infection.

**FIG 3:**
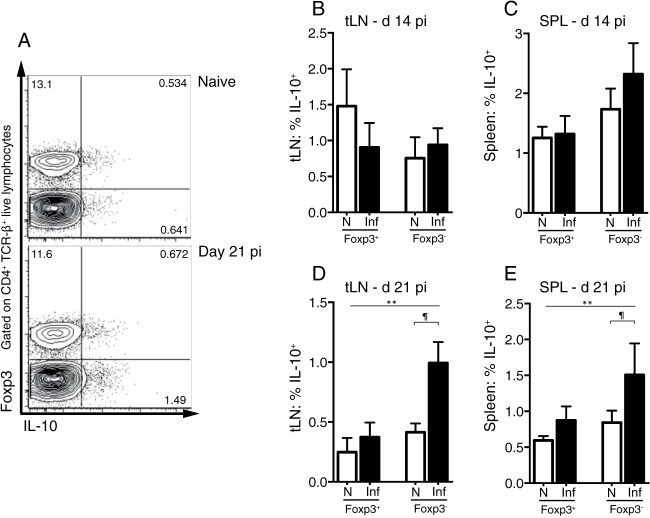
Foxp3^+^ Treg cells do not increase IL-10 production during early-stage S. mansoni infection. C57BL/6 mice were infected with 70 S. mansoni cercariae, and IL-10 production by Foxp3^+^ CD4^+^ TCR-β^+^ T cells and Foxp3^−^ CD4^+^ T cells was measured in the spleen and tLN at days 14 and 21 p.i. (A) Representative staining for Foxp3 and IL-10 production by splenic CD4^+^ T cells. (B and C) Percentages of Foxp3^+^ and Foxp3^−^ IL-10^+^ cells within the CD4^+^ TCR-β^+^ populations at day 14 p.i. in the tLN (B) and spleen (C). (D and E) Percentages of Foxp3^+^ and Foxp3^−^ IL-10^+^ cells within the CD4^+^ TCR-β^+^ populations at day 21 p.i. in the tLN (D) and spleen (E). Panels show combined data from two separate experiments with 4 to 6 mice per group. Bars show mean values, and error bars show standard deviations. Open bars denote results for naive mice, and shaded bars denote results for S. mansoni-infected mice. **, significant effect of infection (*P* < 0.005, ANOVA based on combined data from two independent experiments); ¶, significant pairwise comparison (*P* < 0.05, LSM Student's *t* test).

The suppressive cytokine IL-10 can inhibit vaccine-mediated immunity to S. mansoni (20) and prevents resistance to reinfection (21). To test whether Foxp3^+^ Treg cells produce IL-10 in response to the migration of S. mansoni larvae, we quantified the expression of Foxp3 and IL-10 by flow cytometry of CD4^+^ T cells at days 14 and 21 p.i. (Fig. 3A). Due to low lung cell numbers, our analysis was restricted to the tLN and spleen. There was no increase in the percentage of CD4^+^ Foxp3^+^ Treg cells expressing IL-10 upon infection in either the tLN or spleen (Fig. 3B and C). In contrast to the Foxp3^+^ Treg cell population, at day 21 p.i., there was a significant increase in the percentage of Foxp3^−^ CD4^+^ T cells producing IL-10 in both the tLN and spleen (Fig. 3D and E). Infection did not result in increased IL-10 production by CD4^−^ cells (data not shown). This suggests that Foxp3^+^ Treg cells are not a major source of IL-10 during early S. mansoni infection.

### IL-4-producing Th2 cells predominate in the early phase of infection.

**FIG 4:**
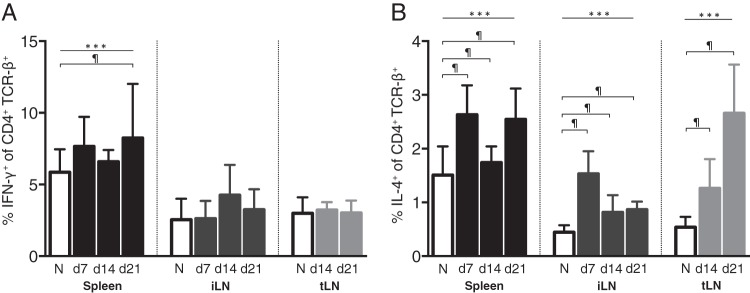
Production of IFN-γ and IL-4 by LN and splenic CD4^+^ T cells during early S. mansoni infection. C57BL/6 mice were infected with 70 S. mansoni cercariae, and the proportions of IFN-γ^+^ (A) and IL-4^+^ (B) cells within the CD4^+^ TCR-β^+^ populations of the spleen, iLN, and tLN were measured by intracellular cytokine staining at days 7, 14, and 21 p.i. Panels show combined data from two separate experiments with 4 to 6 mice per group. Bars show mean values, and error bars show standard deviations. Open bars denote results for naive mice, and shaded bars denote results for S. mansoni-infected mice. ***, significant effect of infection (*P* < 0.0001, ANOVA based on combined data from two independent experiments); ¶, significant pairwise comparison (*P* < 0.05, LSM Student's *t* test).

The importance of the balance of Th1 versus Th2 immune responses during early schistosome infection remains controversial. While vaccine studies originally indicated that the initial immune response to invading S. mansoni larvae is primarily Th1 (18, 29), more recent evidence has shown that Th2 immune responses are also induced at this stage of infection (30). To gain a further understanding of the character of the initial immune response directed against S. mansoni larvae, we measured CD4^+^ T cell IFN-γ and IL-4 production at days 7, 14, and 21 p.i. in the iLN and spleen and at days 14 and 21 p.i. in the tLN by intracellular cytokine staining. Due to limited cell numbers, we were unable to perform intracellular cytokine staining at day 7 p.i. in the tLN. In agreement with previous data (31), we observed significantly increased CD4^+^ T cell IFN-γ production in the spleen at day 21 p.i. (Fig. 4A). However, IFN-γ was not detected at earlier time points (days 7 and 14) in the spleen or at any time p.i. in the tLN and iLN. In contrast, IL-4-expressing T cells increased significantly by day 7 p.i. in the spleen and iLN and remained elevated through days 14 and 21 (Fig. 4B). Similarly, IL-4-expressing T cells increased significantly in the tLN at days 14 and 21 p.i. (Fig. 4B). This, along with the IL-10 data, indicates that the lack of a Foxp3^+^ Treg cell response is not due to a failure to mount an immune response toward the larvae and that, while a mixed Th1 and Th2 response is mounted toward S. mansoni larvae, IL-4-producing Th2 cells predominate during this early period of infection.

### CD4^+^ T cell-derived IL-10 is produced by IL-4^+^ Th2 cells in the early phase of infection.

**FIG 5:**
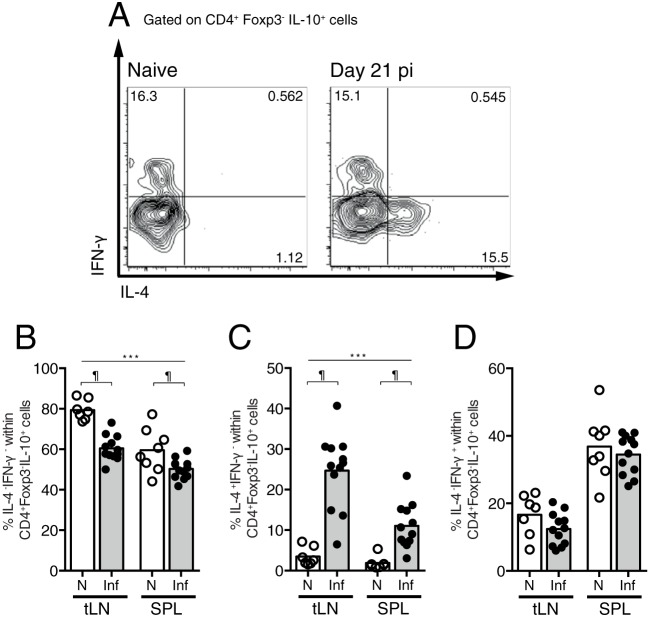
IL-4^+^ Th2 cells are the dominant source of IL-10 during early-stage S. mansoni infection. C57BL/6 mice were infected with 70 S. mansoni cercariae, and the production of IL-4 and IFN-γ by IL-10^+^ Foxp3^−^ CD4^+^ T cells was measured in the spleen and tLN at day 21 p.i. (A) Representative staining for IL-4 and IFN-γ production by tLN IL-10^+^ Foxp3^−^ CD4^+^ T cells. (B to D) Percentages of IL-4^−^ IFN-γ^−^ cells (B), IL-4^+^ IFN-γ^−^ cells (C), and IL-4^−^ IFN-γ^+^ cells (D) within the IL-10^+^ Foxp3^−^ CD4^+^ T cell populations in the tLN and spleen at day 21 p.i. Panels show combined data from two separate experiments with 4 to 6 mice per group. Symbols represent results for individual mice, and bars show mean values. Open circles denote results for naive mice, and closed circles denote results for S. mansoni-infected mice. ***, significant effect of infection (*P* < 0.0001, ANOVA based on combined data from two independent experiments); ¶, significant pairwise comparison (*P* < 0.05, LSM Student's *t* test).

Evidence suggests that during the egg phase of S. mansoni infection, the dominant cellular sources of suppressive IL-10 are either Th2 or Tr1 cells (14, 21, 32, 33). To test the contribution of these cells to the IL-10 produced in the early phase of infection, we measured the proportions of CD4^+^ Foxp3^−^ IL-10^+^ T cells coexpressing IFN-γ and IL-4. The largest population of IL-10 secretors was IL-4^−^ IFN-γ^−^ in both naive and infected mice (Fig. 5A and B). However, the proportion of these cells was significantly reduced upon S. mansoni infection, indicating that S. mansoni infection does not stimulate IL-10^+^ IL-4^−^ IFN-γ^−^ Tr1 cells within the tLN or spleen. Instead, at day 21 p.i., there were significantly increased percentages of IL-4^+^ IFN-γ^−^ cells within the CD4^+^ Foxp3^−^ IL-10^+^ T cell populations in both the tLN and spleen (Fig. 5C) and no change in the proportions of IL-4^−^ IFN-γ^+^ cells (Fig. 5D). Similarly, there was an increase in the total numbers of CD4^+^ Foxp3^−^ IL-10^+^ IL-4^+^ IFN-γ^−^ T cells but no change in the total numbers of CD4^+^ Foxp3^−^ IL-10^+^ IL-4^−^ IFN-γ^+^ or CD4^+^ Foxp3^−^ IL-10^+^ IL-4^−^ IFN-γ^−^ T cells (data not shown). These data indicate that IL-4^+^ Th2 cells increase their production of IL-10 in the LN and spleen during early S. mansoni infection.

## DISCUSSION

Schistosomiasis is one of the most important neglected tropical diseases, with over 200 million people afflicted in many low- and middle-income countries (34). Chronic infection has been associated with increased immunoregulatory mechanisms, including the expansion of regulatory T cells (35, 36) and the dampening of allergic and autoimmune responses to bystander antigens (37–40). Rapid expansion of Foxp3^+^ Treg cells has been clearly shown to occur in the early phase of several helminth infections, such as nematode parasites in mouse models, resulting in impaired protective immunity. Foxp3^+^ Treg cells rapidly become activated and increase in number during the larval stages of Litomosoides sigmodontis infection, and ablation of CD25^+^ Treg cells before or during infection enhances parasite killing (7, 25, 41). Similarly, early expansion of Foxp3^+^ Treg cells that inhibit type 2 immunity is observed during intestinal nematode infections with H. polygyrus, Trichuris muris, and Strongyloides ratti (8, 9, 11, 24, 42). In S. ratti and T. muris infections, Foxp3^+^ Treg cells act very early, and protective immunity can only be restored if they are depleted prior to day 4 or day 9 of infection, respectively (8, 11, 24). Hence, we investigated whether S. mansoni parasites would also induce Foxp3^+^ Treg cell expansion during the early stages of infection when the parasites transit the lungs. However, our study demonstrates that, in contrast to the response to other helminths, Foxp3^+^ Treg cells do not expand, upregulate activation markers, or produce IL-10 during the early larval stages of S. mansoni infection in C57BL/6 mice.

Altogether, this suggests that migrating S. mansoni larvae do not initiate a Foxp3^+^ Treg cell response in C57BL/6 mice and that co-option of Treg cell function is not a survival strategy used by S. mansoni parasites to avoid host immune attack. In keeping with this, depletion of CD25^+^ T cells immediately prior to S. mansoni infection of C57BL/6 mice does not affect the parasite burden (15). Although overt Foxp3^+^ Treg cell activity was not detected, it is possible that Foxp3^+^ Treg cells are playing a subtle role in regulating early immunity to S. mansoni larvae, and functional studies are required to confirm this. The lack of Foxp3^+^ Treg cell expansion during the larval stages of infection in C57BL/6 mice contrasts with the evident Foxp3^+^ Treg cell activity during the chronic phase of infection, following egg release (week 5 onwards) (14–17), although it is not known whether Foxp3^+^ Treg cells are recruited to or activated in the lungs during pulmonary schistosomiasis. Similarly, in BALB/c mice, there is little evidence of Foxp3^+^ Treg cell expansion at week 4 p.i., prior to egg release (43). An interesting future direction for investigation will be to determine whether only S. mansoni eggs and not S. mansoni parasites stimulate Foxp3^+^ Treg cells.

Exposure to S. mansoni egg antigens induces Foxp3 expression in CD4^+^ T cells from NOD mice but not C57BL/6 mice (44). Similarly, depletion of Foxp3^+^ Treg cells increases resistance to S. ratti in BALB/c mice but not in C57BL/6 mice (24). Thus, the importance of Foxp3^+^ Treg cell responses during helminth infections can differ depending on the mouse strain, with Foxp3^+^ Treg cells potentially playing a lesser role in C57BL/6 mice. However, C57BL/6 mice are capable of mounting strong Foxp3^+^ Treg cell responses to helminths, as rapid Foxp3^+^ Treg cell responses are seen in response to filarial nematodes (7) and Foxp3^+^ Treg cell depletion promotes resistance to T. muris (11). Thus, while it is likely that the lack of Foxp3^+^ Treg cell activity in early S. mansoni infection is a general feature of infection, there remains the possibility that in other, as yet untested strains, Foxp3^+^ Treg cell activation and expansion may occur.

S. mansoni employs a number of strategies for defense against host immunity, such that it may not need to utilize Foxp3^+^ Treg cells for this purpose. For example, S. mansoni parasites have acquired the ability to hide from the host's immune system, secreting a membranocalyx that surrounds them and shields vulnerable surface proteins from immune recognition (45). Embedded in the membranocalyx are blood group antigens and host major histocompatibility complex (MHC) molecules that disguise the parasites from immune attack (46, 47).

At the cellular level, widespread interactions occur between schistosomes and host immune populations. Thus, larval secretions generate a population of antigen-presenting cells that prime CD4^+^ T cells to produce high levels of the suppressive cytokine IL-10 (48). In addition, IL-10 from a range of different cell types, including regulatory B cells and innate effector cells, is important in chronic schistosomiasis (49–52), but to date, no evidence of their activation in the early larval stage of infection has been obtained.

In contrast, early CD4^+^ T cell IL-10 expression is observed during vaccination settings, in which radiation-attenuated S. mansoni larvae stimulate the production of IL-10, which suppresses the development of a robust Th1 response in the skin-draining lymph nodes (53, 54). The induction of Th1 cells in the skin-draining lymph nodes is required for their recruitment to the lung (18), where they initiate the formation of inflammatory foci which block the passage of transiting larvae (19), and in the absence of IL-10, protective immunity to S. mansoni infection is enhanced (20). Similarly, IL-10 prevents the development of protective memory responses to S. mansoni larvae (21). This suggests that the induction of IL-10 is a key survival strategy used by S. mansoni larvae for evasion of host immunity.

While IL-10 impairs protective immunity during the early stages of S. mansoni infection (20), during the egg phase of disease, IL-10 acts primarily to suppress immunopathology (55). Although Foxp3^+^ Treg cells are a major source of IL-10 at the site of H. polygyrus infection (10), evidence suggests that the majority of T cell-derived IL-10 produced during the patent egg phase of S. mansoni infection comes from Foxp3^−^ Th2 cells (14, 21, 33), although there is some evidence for the induction of Tr1 cells (32). Similarly, CD4^+^ Foxp3^−^ T cells are the main T cell source of IL-10 in other filarial and intestinal helminth infections (56–58). We observed that the largest population of IL-10-producing CD4^+^ T cells in both naive and infected mice was negative for Foxp3, IL-4, and IFN-γ. However, during early S. mansoni infection, the proportion of these cells declined and a population of IL-10^+^ Foxp3^−^ CD4^+^ IL-4^+^ IFN-γ^−^ Th2 cells emerged. This suggests that Th2 cells are the main T cell population stimulated to produce IL-10 during both the lung and egg stage of S. mansoni infection. Therefore, the lack of an early lung-stage Foxp3^+^ Treg cell response could reflect the fact that non-Treg, Th2 cell-derived IL-10 plays a suppressive role, and S. mansoni does not need to elicit a Foxp3^+^ Treg cell response to evade immune attack.

In summary, this work has demonstrated that the character of the initial immune response invoked by S. mansoni parasites in C57BL/6 mice contrasts with the responses to other parasitic helminth infections, which promote rapid Foxp3^+^ Treg cell responses. Thus, S. mansoni larvae do not cause the activation of, expansion of, or IL-10 production by Foxp3^+^ Treg cells during the early migratory phase of infection. This suggests that Foxp3^+^ Treg cells do not play a major role in regulating immunity to schistosome larval stages and that co-option of Treg cell function is not a survival mechanism employed by this trematode parasite.

## References

[1] Maizels RM, Balic A, Gomez-Escobar N, Nair M, Taylor MD, Allen JE 2004 Helminth parasites—masters of regulation. Immunol Rev 201:89–116. doi:10.1111/j.0105-2896.2004.00191.x. 15361235

[2] Maizels RM, Yazdanbakhsh M 2003 Immune regulation by helminth parasites: cellular and molecular mechanisms. Nat Rev Immunol 3:733–744. doi:10.1038/nri1183. 12949497

[3] Vignali DA, Collison LW, Workman CJ 2008 How regulatory T cells work. Nat Rev Immunol 8:523–532. doi:10.1038/nri2343. 18566595 PMC2665249

[4] Taylor MD, van der Werf N, Maizels RM 2012 T cells in helminth infection: the regulators and the regulated. Trends Immunol 33:181–189. doi:10.1016/j.it.2012.01.001. 22398370

[5] Gillan V, Devaney E 2005 Regulatory T cells modulate Th2 responses induced by Brugia pahangi third-stage larvae. Infect Immun 73:4034–4042. doi:10.1128/IAI.73.7.4034-4042.2005. 15972491 PMC1168597

[6] McSorley HJ, Harcus YM, Murray J, Taylor MD, Maizels RM 2008 Expansion of Foxp3^+^ regulatory T cells in mice infected with the filarial parasite Brugia malayi. J Immunol 181:6456–6466. doi:10.4049/jimmunol.181.9.6456. 18941236

[7] Taylor MD, van der Werf N, Harris A, Graham AL, Bain O, Allen JE, Maizels RM 2009 Early recruitment of natural CD4^+^ Foxp3^+^ Treg cells by infective larvae determines the outcome of filarial infection. Eur J Immunol 39:192–206. doi:10.1002/eji.200838727. 19089814

[8] Blankenhaus B, Klemm U, Eschbach M-L, Sparwasser T, Huehn J, Kühl AA, Loddenkemper C, Jacobs T, Breloer M 2011 Strongyloides ratti infection induces expansion of Foxp3^+^ regulatory T cells that interfere with immune response and parasite clearance in BALB/c mice. J Immunol 186:4295–4305. doi:10.4049/jimmunol.1001920. 21335490

[9] Finney CA, Taylor MD, Wilson MS, Maizels RM 2007 Expansion and activation of CD4^+^CD25^+^ regulatory T cells in Heligmosomoides polygyrus infection. Eur J Immunol 37:1874–1886. doi:10.1002/eji.200636751. 17563918 PMC2699425

[10] Redpath SA, van der Werf N, Cervera AM, MacDonald AS, Gray D, Maizels RM, Taylor MD 2013 ICOS controls Foxp3^+^ regulatory T-cell expansion, maintenance and IL-10 production during helminth infection. Eur J Immunol 43:705–715. doi:10.1002/eji.201242794. 23319295 PMC3615169

[11] Sawant DV, Gravano DM, Vogel P, Giacomin P, Artis D, Vignali DA 2014 Regulatory T cells limit induction of protective immunity and promote immune pathology following intestinal helminth infection. J Immunol 192:2904–2912. doi:10.4049/jimmunol.1202502. 24532574 PMC3955731

[12] Gryseels B, Polman K, Clerinx J, Kestens L 2006 Human schistosomiasis. Lancet 368:1106–1118. doi:10.1016/S0140-6736(06)69440-3. 16997665

[13] Pearce EJ, MacDonald AS 2002 The immunobiology of schistosomiasis. Nat Rev Immunol 2:499–511. doi:10.1038/nri843. 12094224

[14] Baumgart M, Tompkins F, Leng J, Hesse M 2006 Naturally occurring CD4^+^Foxp3^+^ regulatory T cells are an essential, IL-10-independent part of the immunoregulatory network in Schistosoma mansoni egg-induced inflammation. J Immunol 176:5374–5387. doi:10.4049/jimmunol.176.9.5374. 16622005

[15] Layland LE, Rad R, Wagner H, da Costa CUP 2007 Immunopathology in schistosomiasis is controlled by antigen-specific regulatory T cells primed in the presence of TLR2. Eur J Immunol 37:2174–2184. doi:10.1002/eji.200737063. 17621370

[16] Singh KP, Gerard HC, Hudson AP, Reddy TR, Boros DL 2005 Retroviral Foxp3 gene transfer ameliorates liver granuloma pathology in Schistosoma mansoni infected mice. Immunology 114:410–417. doi:10.1111/j.1365-2567.2004.02083.x. 15720442 PMC1782091

[17] Turner JD, Jenkins GR, Hogg KG, Aynsley SA, Paveley RA, Cook PC, Coles MC, Mountford AP 2011 CD4^+^CD25^+^ regulatory cells contribute to the regulation of colonic Th2 granulomatous pathology caused by schistosome infection. PLoS Negl Trop Dis 5:e1269. doi:10.1371/journal.pntd.0001269. 21858239 PMC3153428

[18] Mountford AP, Coulson PS, Pemberton RM, Smythies LE, Wilson RA 1992 The generation of interferon-gamma-producing T lymphocytes in skin-draining lymph nodes, and their recruitment to the lungs, is associated with protective immunity to Schistosoma mansoni. Immunology 75:250–256. 1532378 PMC1384702

[19] Smythies LE, Coulson PS, Wilson RA 1992 Monoclonal antibody to IFN-gamma modifies pulmonary inflammatory responses and abrogates immunity to Schistosoma mansoni in mice vaccinated with attenuated cercariae. J Immunol 149:3654–3658. 1431135

[20] Hoffmann KF, James SL, Cheever AW, Wynn TA 1999 Studies with double cytokine-deficient mice reveal that highly polarized Th1- and Th2-type cytokine and antibody responses contribute equally to vaccine-induced immunity to Schistosoma mansoni. J Immunol 163:927–938. 10395689

[21] Wilson MS, Cheever AW, White SD, Thompson RW, Wynn TA 2011 IL-10 blocks the development of resistance to re-infection with Schistosoma mansoni. PLoS Pathog 7:e1002171. doi:10.1371/journal.ppat.1002171. 21829367 PMC3150278

[22] Angeli V, Faveeuw C, Delerive P, Fontaine J, Barriera Y, Franchimont N, Staels B, Capron M, Trottein F 2001 Schistosoma mansoni induces the synthesis of IL-6 in pulmonary microvascular endothelial cells: role of IL-6 in the control of lung eosinophilia during infection. Eur J Immunol 31:2751–2761. doi:10.1002/1521-4141(200109)31:9<2751::AID-IMMU2751>3.0.CO;2-4. 11536174

[23] Smith KA, Maizels RM 2014 IL-6 controls susceptibility to helminth infection by impeding Th2 responsiveness and altering the Treg phenotype *in vivo*. Eur J Immunol 44:150–161. doi:10.1002/eji.201343746. 24185641 PMC3992848

[24] Blankenhaus B, Reitz M, Brenz Y, Eschbach ML, Hartmann W, Haben I, Sparwasser T, Huehn J, Kuhl A, Feyerabend TB, Rodewald HR, Breloer M 2014 Foxp3^+^ regulatory T cells delay expulsion of intestinal nematodes by suppression of IL-9-driven mast cell activation in BALB/c but not in C57BL/6 mice. PLoS Pathog 10:e1003913. doi:10.1371/journal.ppat.1003913. 24516385 PMC3916398

[25] Taylor MD, LeGoff L, Harris A, Malone E, Allen JE, Maizels RM 2005 Removal of regulatory T cell activity reverses hyporesponsiveness and leads to filarial parasite clearance *in vivo*. J Immunol 174:4924–4933. doi:10.4049/jimmunol.174.8.4924. 15814720

[26] Rausch S, Huehn J, Kirchhoff D, Rzepecka J, Schnoeller C, Pillai S, Loddenkemper C, Scheffold A, Hamann A, Lucius R, Hartmann S 2008 Functional analysis of effector and regulatory T cells in a parasitic nematode infection. Infect Immun 76:1908–1919. doi:10.1128/IAI.01233-07. 18316386 PMC2346705

[27] Thornton AM, Korty PE, Tran DQ, Wohlfert EA, Murray PE, Belkaid Y, Shevach EM 2010 Expression of Helios, an Ikaros transcription factor family member, differentiates thymic-derived from peripherally induced Foxp3^+^ T regulatory cells. J Immunol 184:3433–3441. doi:10.4049/jimmunol.0904028. 20181882 PMC3725574

[28] Akimova T, Beier UH, Wang L, Levine MH, Hancock WW 2011 Helios expression is a marker of T cell activation and proliferation. PLoS One 6:e24226. doi:10.1371/journal.pone.0024226. 21918685 PMC3168881

[29] Smythies LE, Pemberton RM, Coulson PS, Mountford AP, Wilson RA 1992 T cell-derived cytokines associated with pulmonary immune mechanisms in mice vaccinated with irradiated cercariae of Schistosoma mansoni. J Immunol 148:1512–1518. 1538133

[30] Cook PC, Aynsley SA, Turner JD, Jenkins GR, Van Rooijen N, Leeto M, Brombacher F, Mountford AP 2011 Multiple helminth infection of the skin causes lymphocyte hypo-responsiveness mediated by Th2 conditioning of dermal myeloid cells. PLoS Pathog 7:e1001323. doi:10.1371/journal.ppat.1001323. 21445234 PMC3060168

[31] Pearce EJ, Caspar P, Grzych JM, Lewis FA, Sher A 1991 Downregulation of Th1 cytokine production accompanies induction of Th2 responses by a parasitic helminth, Schistosoma mansoni. J Exp Med 173:159–166. doi:10.1084/jem.173.1.159. 1824635 PMC2118762

[32] Freeman CM, Chiu BC, Stolberg VR, Hu J, Zeibecoglou K, Lukacs NW, Lira SA, Kunkel SL, Chensue SW 2005 CCR8 is expressed by antigen-elicited, IL-10-producing CD4^+^CD25^+^ T cells, which regulate Th2-mediated granuloma formation in mice. J Immunol 174:1962–1970. doi:10.4049/jimmunol.174.4.1962. 15699124 PMC1599789

[33] Taylor JJ, Mohrs M, Pearce EJ 2006 Regulatory T cell responses develop in parallel to Th responses and control the magnitude and phenotype of the Th effector population. J Immunol 176:5839–5847. doi:10.4049/jimmunol.176.10.5839. 16670290

[34] Colley DG, Bustinduy AL, Secor WE, King CH 2014 Human schistosomiasis. Lancet 383:2253–2264. doi:10.1016/S0140-6736(13)61949-2. 24698483 PMC4672382

[35] Teixeira-Carvalho A, Martins-Filho OA, Peruhype-Magalhaes V, Silveira-Lemos D, Malaquias LC, Oliveira LF, Silveira AM, Gazzinelli A, Gazzinelli G, Correa-Oliveira R 2008 Cytokines, chemokine receptors, CD4^+^CD25^high+^ T-cells and clinical forms of human schistosomiasis. Acta Trop 108:139–149. doi:10.1016/j.actatropica.2008.04.010. 18534548

[36] Watanabe K, Mwinzi PN, Black CL, Muok EM, Karanja DM, Secor WE, Colley DG 2007 T regulatory cell levels decrease in people infected with Schistosoma mansoni on effective treatment. Am J Trop Med Hyg 77:676–682. 17978070 PMC2602861

[37] Araujo MI, de Carvalho EM 2006 Human schistosomiasis decreases immune responses to allergens and clinical manifestations of asthma. Chem Immunol Allergy 90:29–44. 16210901 10.1159/000088879

[38] Mutapi F, Imai N, Nausch N, Bourke CD, Rujeni N, Mitchell KM, Midzi N, Woolhouse ME, Maizels RM, Mduluza T 2011 Schistosome infection intensity is inversely related to auto-reactive antibody levels. PLoS One 6:e19149. doi:10.1371/journal.pone.0019149. 21573157 PMC3089602

[39] Rujeni N, Nausch N, Bourke CD, Midzi N, Mduluza T, Taylor DW, Mutapi F 2012 Atopy is inversely related to schistosome infection intensity: a comparative study in Zimbabwean villages with distinct levels of Schistosoma haematobium infection. Int Arch Allergy Immunol 158:288–298. doi:10.1159/000332949. 22398631 PMC3398828

[40] van den Biggelaar AH, van Ree R, Rodrigues LC, Lell B, Deelder AM, Kremsner PG, Yazdanbakhsh M 2000 Decreased atopy in children infected with Schistosoma haematobium: a role for parasite-induced interleukin-10. Lancet 356:1723–1727. doi:10.1016/S0140-6736(00)03206-2. 11095260

[41] Taylor MD, Harris A, Babayan SA, Bain O, Culshaw A, Allen JE, Maizels RM 2007 CTLA-4 and CD4^+^ CD25^+^ regulatory T cells inhibit protective immunity to filarial parasites *in vivo*. J Immunol 179:4626–4634. doi:10.4049/jimmunol.179.7.4626. 17878360

[42] Rausch S, Huehn J, Loddenkemper C, Hepworth M, Klotz C, Sparwasser T, Hamann A, Lucius R, Hartmann S 2009 Establishment of nematode infection despite increased Th2 responses and immunopathology after selective depletion of Foxp3^+^ cells. Eur J Immunol 39:3066–3077. doi:10.1002/eji.200939644. 19750483

[43] Walsh CM, Smith P, Fallon PG 2007 Role for CTLA-4 but not CD25^+^ T cells during Schistosoma mansoni infection of mice. Parasite Immunol 29:293–308. doi:10.1111/j.1365-3024.2007.00947.x. 17518948

[44] Zaccone P, Burton O, Miller N, Jones FM, Dunne DW, Cooke A 2009 Schistosoma mansoni egg antigens induce Treg that participate in diabetes prevention in NOD mice. Eur J Immunol 39:1098–1107. doi:10.1002/eji.200838871. 19291704

[45] Wilson RA, Coulson PS 2009 Immune effector mechanisms against schistosomiasis: looking for a chink in the parasite's armour. Trends Parasitol 25:423–431. doi:10.1016/j.pt.2009.05.011. 19717340 PMC3686490

[46] McLaren DJ, Terry RJ 1982 The protective role of acquired host antigens during schistosome maturation. Parasite Immunol 4:129–148. doi:10.1111/j.1365-3024.1982.tb00426.x. 7070835

[47] Sher A, Hall BF, Vadas MA 1978 Acquisition of murine major histocompatibility complex gene products by schistosomula of Schistosoma mansoni. J Exp Med 148:46–57. doi:10.1084/jem.148.1.46. 97360 PMC2184927

[48] Jenkins SJ, Mountford AP 2005 Dendritic cells activated with products released by schistosome larvae drive Th2-type immune responses, which can be inhibited by manipulation of CD40 costimulation. Infect Immun 73:395–402. doi:10.1128/IAI.73.1.395-402.2005. 15618177 PMC538949

[49] Mangan NE, Fallon RE, Smith P, van Rooijen N, McKenzie AN, Fallon PG 2004 Helminth infection protects mice from anaphylaxis via IL-10-producing B cells. J Immunol 173:6346–6356. doi:10.4049/jimmunol.173.10.6346. 15528374

[50] Smits HH, Hammad H, van Nimwegen M, Soullie T, Willart MA, Lievers E, Kadouch J, Kool M, Kos-van Oosterhoud J, Deelder AM, Lambrecht BN, Yazdanbakhsh M 2007 Protective effect of Schistosoma mansoni infection on allergic airway inflammation depends on the intensity and chronicity of infection. J Allergy Clin Immunol 120:932–940. doi:10.1016/j.jaci.2007.06.009. 17689595

[51] van der Vlugt LE, Labuda LA, Ozir-Fazalalikhan A, Lievers E, Gloudemans AK, Liu KY, Barr TA, Sparwasser T, Boon L, Ngoa UA, Feugap EN, Adegnika AA, Kremsner PG, Gray D, Yazdanbakhsh M, Smits HH 2012 Schistosomes induce regulatory features in human and mouse CD1d^hi^ B cells: inhibition of allergic inflammation by IL-10 and regulatory T cells. PLoS One 7:e30883. doi:10.1371/journal.pone.0030883. 22347409 PMC3275567

[52] Hesse M, Piccirillo CA, Belkaid Y, Prufer J, Mentink-Kane M, Leusink M, Cheever AW, Shevach EM, Wynn TA 2004 The pathogenesis of schistosomiasis is controlled by cooperating IL-10-producing innate effector and regulatory T cells. J Immunol 172:3157–3166. doi:10.4049/jimmunol.172.5.3157. 14978122

[53] Hogg KG, Kumkate S, Mountford AP 2003 IL-10 regulates early IL-12-mediated immune responses induced by the radiation-attenuated schistosome vaccine. Int Immunol 15:1451–1459. doi:10.1093/intimm/dxg142. 14645154

[54] Hogg KG, Kumkate S, Anderson S, Mountford AP 2003 Interleukin-12 p40 secretion by cutaneous CD11c^+^ and F4/80^+^ cells is a major feature of the innate immune response in mice that develop Th1-mediated protective immunity to Schistosoma mansoni. Infect Immun 71:3563–3571. doi:10.1128/IAI.71.6.3563-3571.2003. 12761141 PMC155763

[55] Hoffmann KF, Cheever AW, Wynn TA 2000 IL-10 and the dangers of immune polarization: excessive type 1 and type 2 cytokine responses induce distinct forms of lethal immunopathology in murine schistosomiasis. J Immunol 164:6406–6416. doi:10.4049/jimmunol.164.12.6406. 10843696

[56] Beiting DP, Gagliardo LF, Hesse M, Bliss SK, Meskill D, Appleton JA 2007 Coordinated control of immunity to muscle stage Trichinella spiralis by IL-10, regulatory T cells, and TGF-beta. J Immunol 178:1039–1047. doi:10.4049/jimmunol.178.2.1039. 17202367

[57] Metenou S, Dembele B, Konate S, Dolo H, Coulibaly SY, Coulibaly YI, Diallo AA, Soumaoro L, Coulibaly ME, Sanogo D, Doumbia SS, Traore SF, Mahanty S, Klion A, Nutman TB 2010 At homeostasis filarial infections have expanded adaptive T regulatory but not classical Th2 cells. J Immunol 184:5375–5382. doi:10.4049/jimmunol.0904067. 20357251 PMC3407820

[58] Mitre E, Chien D, Nutman TB 2008 CD4^+^ (and not CD25^+^) T cells are the predominant interleukin-10-producing cells in the circulation of filaria-infected patients. J Infect Dis 197:94–101. doi:10.1086/524301. 18171291

